# Competing endogenous RNA (ceRNA) networks in Parkinson's disease: A systematic review

**DOI:** 10.3389/fncel.2023.1044634

**Published:** 2023-01-24

**Authors:** Mohammad Reza Asadi, Samin Abed, Ghazal Kouchakali, Fateme Fattahi, Hani Sabaie, Marziyeh Sadat Moslehian, Mirmohsen Sharifi-Bonab, Bashdar Mahmud Hussen, Mohammad Taheri, Soudeh Ghafouri-Fard, Maryam Rezazadeh

**Affiliations:** ^1^Clinical Research Development Unit of Tabriz Valiasr Hospital, Tabriz University of Medical Sciences, Tabriz, Iran; ^2^Department of Medical Genetics, Faculty of Medicine, Tabriz University of Medical Sciences, Tabriz, Iran; ^3^Department of Biomedical Sciences, Cihan University-Erbil, Erbil, Iraq; ^4^Department of Pharmacognosy, College of Pharmacy, Hawler Medical University, Erbil, Iraq; ^5^Urology and Nephrology Research Center, Shahid Beheshti University of Medical Sciences, Tehran, Iran; ^6^Institute of Human Genetics, Jena University Hospital, Jena, Germany; ^7^Department of Medical Genetics, Shahid Beheshti University of Medical Sciences, Tehran, Iran

**Keywords:** Parkinson's disease, ceRNA, miRNA, lncRNA, circRNA, lincRNA, NEAT1, SNHG

## Abstract

Parkinson's disease (PD) is a distinctive clinical syndrome with several causes and clinical manifestations. Aside from an infectious cause, PD is a rapidly developing neurological disorder with a global rise in frequency. Notably, improved knowledge of molecular pathways and the developing novel diagnostic methods may result in better therapy for PD patients. In this regard, the amount of research on ceRNA axes is rising, highlighting the importance of these axes in PD. CeRNAs are transcripts that cross-regulate one another *via* competition for shared microRNAs (miRNAs). These transcripts may be either coding RNAs (mRNAs) or non-coding RNAs (ncRNAs). This research used a systematic review to assess validated loops of ceRNA in PD. The Prisma guideline was used to conduct this systematic review, which entailed systematically examining the articles of seven databases. Out of 309 entries, forty articles met all criteria for inclusion and were summarized in the appropriate table. CeRNA axes have been described through one of the shared vital components of the axes, including lncRNAs such as NEAT1, SNHG family, HOTAIR, MALAT1, XIST, circRNAs, and lincRNAs. Understanding the multiple aspects of this regulatory structure may aid in elucidating the unknown causal causes of PD and providing innovative molecular therapeutic targets and medical fields.

## 1. Introduction

Parkinson's disease (PD) is a neurological disorder that affects over 6 million individuals worldwide and whose incidence is anticipated to double by 2040 (Dorsey et al., 2018a,b). It is distinguished by a core set of movement (motor) problems, including slowness of movement, muscular stiffness, and tremors, as well as a variety of non-motor symptoms, including constipation, anxiety, and dementia (Blochberger and Jones, 2011). A prodromal period of non-motor symptoms often precedes motor symptoms by several years (Sebastian et al., 2019). The main pathologic feature of PD is the presence of Lewy bodies, which are clumps of misfolded α-synuclein protein (encoded by the SNCA gene) that cause the loss of dopamine-producing neurons in the midbrain. However, the only diagnostic criteria for PD are clinical symptoms (Hughes et al., 1992; Tomlinson et al., 2010). As a result, Parkinsonism may be regarded as a clinical condition with several aetiological paths that lead to the ultimate shared presentation of dopamine depletion and clinical Parkinsonism. There are currently no disease-modifying medications for PD and therapy is primarily focused on dopamine replacement to ameliorate symptoms. The most significant risk factor for PD development is age, and males are more vulnerable than women, with a prevalence ratio of around 3:2. Disease risk has a high hereditary component, with over 90 genetic risk loci already identified (Blauwendraat et al., 2020). Furthermore, PD often manifests sporadically; however, 15.7% of patients report an afflicted relative of the first degree (Bentley et al., 2018). Furthermore, some potentially modifiable environmental (e.g., pesticides, water pollutants) and behavioral variables (e.g., use of cigarettes, coffee, exercise, or head trauma) (Feigin and Collaborators, 2019) have been stated to have a role in the etiology of PD in various populations. While breakthroughs in etiology and epidemiology have been outstanding (Dickson et al., 2009; Poewe et al., 2017), the primary cause and fundamental mechanism of PD remain unknown, and no cure or preventative medication has yet been discovered. In this respect, focusing on the molecular mechanisms that have the potential to play a critical role in PD can be considered. The competing endogenous RNA (ceRNA) axis is one of the possible molecular mechanisms that attract much attention in PD.

CeRNAs are transcripts composed of messenger RNA (mRNAs) and non-coding RNAs (ncRNAs) that contest for shared microRNAs (miRNAs) to cross-regulate one another (Asadi et al., 2022). Franco-Zorrilla et al. reported a process known as “target mimicry,” which occurs when a non-coding RNA in plants sequesters miR-399 and de-represses its target (Franco-Zorrilla et al., 2007). Ebert et al. identified a similar phenomenon in animal cells not long after. Ectopic production of a miRNA with a large number of binding sites [also known as miRNA response elements (MREs)] leads to barely visible miRNA sequestration but a 1.5–2.5-fold overexpression of the miRNA's targets in this study (Ebert et al., 2007). Consequently, the term “RNA sponge” was coined to describe the process of miRNAs being absorbed by overexpressed MRE-containing transcripts. Afterward, the RNA sponge process was discovered in various cancers (Selbach et al., 2008; Poliseno et al., 2010). In 2011, the word “ceRNA” was coined to describe this extra layer of post-transcriptional regulation, which includes mRNAs and ncRNAs (Salmena et al., 2011).

ncRNAs are classified into two types depending on their length: small ncRNAs (200 nucleotides) and long ncRNAs (>200 nucleotides) (Amin et al., 2019). MiRNAs, around 22 nucleotides long and regulating gene expression post-transcriptionally in a sequence-specific way, stand out among short ncRNAs (Singh and Storey, 2021). Approximately 70% of the known miRNAs are expressed in the brain (Cao et al., 2006), and they have been regarded as critical regulators of neuronal homeostasis, with their dysregulation linked to CNS pathology (Quinlan et al., 2017). Long ncRNAs (lncRNAs) are the most abundant ncRNAs in the mammalian genome, and they are further subdivided into linear RNAs and circular RNAs (Amin et al., 2019). Linear lncRNAs (referred to as lncRNAs) exhibit transcriptional and post-transcriptional activity comparable to protein-coding mRNA (Amin et al., 2019). However, lncRNAs have a distinct biological function from mRNAs. Furthermore, they have been linked to brain development, neuronal function, maintenance, and differentiation (Asadi et al., 2021). Circular RNAs (circRNAs) are a relatively new family of RNAs distinguished by a covalent connection that connects the 5′ and 3′ ends and offers more remarkable persistence (half-life of 48 vs. 10 h for mRNAs) (Jeck et al., 2013). CircRNAs are prevalent in the brain, are abundant in synaptoneurosomes, and are increased during neuronal development (Rybak-Wolf et al., 2015). Furthermore, despite being considered dormant gene sequences, a significant fraction of pseudogenes may be translated into ncRNAs (Pei et al., 2012; Guo et al., 2014). Indeed, there is accumulating evidence that pseudogenes might influence the expression of both parental and unrelated genes (Pei et al., 2012; Guo et al., 2014). As a result, altering pseudogene transcription might disrupt gene expression homeostasis, resulting in disease (Guo et al., 2014).

Surprisingly, all of the ncRNAs stated above can be integrated into the ceRNA axis, and these ncRNA components have been examined in the form of ceRNA networks in several studies. Because ceRNA interaction networks are multifactorial, they may help identify therapeutic targets for complex disorders like PD. By targeting only one of them, numerous disease-related RNA levels simultaneously alter (Moreno-García et al., 2020). In this work, we conducted a systematic review to investigate the possibility of verified ceRNA loops in PD. Our research focused on the ceRNA axis, which has been linked to PD pathogenesis and might be used as a therapeutic target.

## 2. Methods

### 2.1. Search strategy

The present systematic review was carried out in accordance with the PRISMA guideline (Moher et al., 2010). The following electronic databases were searched without constraints in order to find all published research up to August 26, 2022: PubMed, Embase, Scopus, Web of Science, and Cochrane, utilizing keywords, MeSH, or Emtree terms discovered in the original search. Google Scholar and ProQuest searched for unpublished research and gray literature. In a systematic PubMed search for PD, the crucial keywords were “idiopathic Parkinson's disease,” “Parkinson^*^,” “primary Parkinsonism,” “Parkinsonian disorder,” “Parkinsonian syndrome,” “paralysis agitans,” and “Lewy body.”

### 2.2. Study selection and assessment of studies

Following the database search, all identified studies were imported into Endnote Version 20.2.1, and duplicates were eliminated. The remaining studies' titles and abstracts were reviewed, and all ceRNA axes in PD were included based on our inclusion criteria. The included publications satisfied the following criteria: they were (1) explicitly describing the ceRNA axis in PD, (2) published in English, and (3) original research. The following research was excluded: (1) non-PD or any neurodegenerative disease studies; (2) studies that did not include human specimens, cell lines, or animal models; and (3) studies that did not use a molecular method to validate the ceRNA loop components.

### 2.3. Data extraction

A self-conducted data extraction technique was used to obtain the necessary data from the research. It listed the authors, the year of publication, the origin, the type of study, human samples, cell lines, animal models, ceRNA(s), shared miRNA(s), major methods, and the major findings.

### 2.4. Bioinformatics analysis

Protein-protein interactions (PPI) were established based on target genes in the ceRNA axes in PD utilizing the Search Tool for the Retrieval of Interacting Genes (STRING) database (http://string-db.org/), which is a web-based tool for evaluating PPI data (Szklarczyk et al., 2021). Gene ontology (GO) enrichment analysis is a frequently used technique for assessing functional enrichment in high-throughput genomic or transcriptomic data. GO terminology is divided into biological process, molecular function, and cellular component. EnrichR, a web-based tool that gives various types of graphical representations of the collective functions of gene lists (Kuleshov et al., 2016), was utilized in this investigation to perform GO function enrichment analysis for ceRNA axis target genes.

## 3. Results

**Figure 1:**
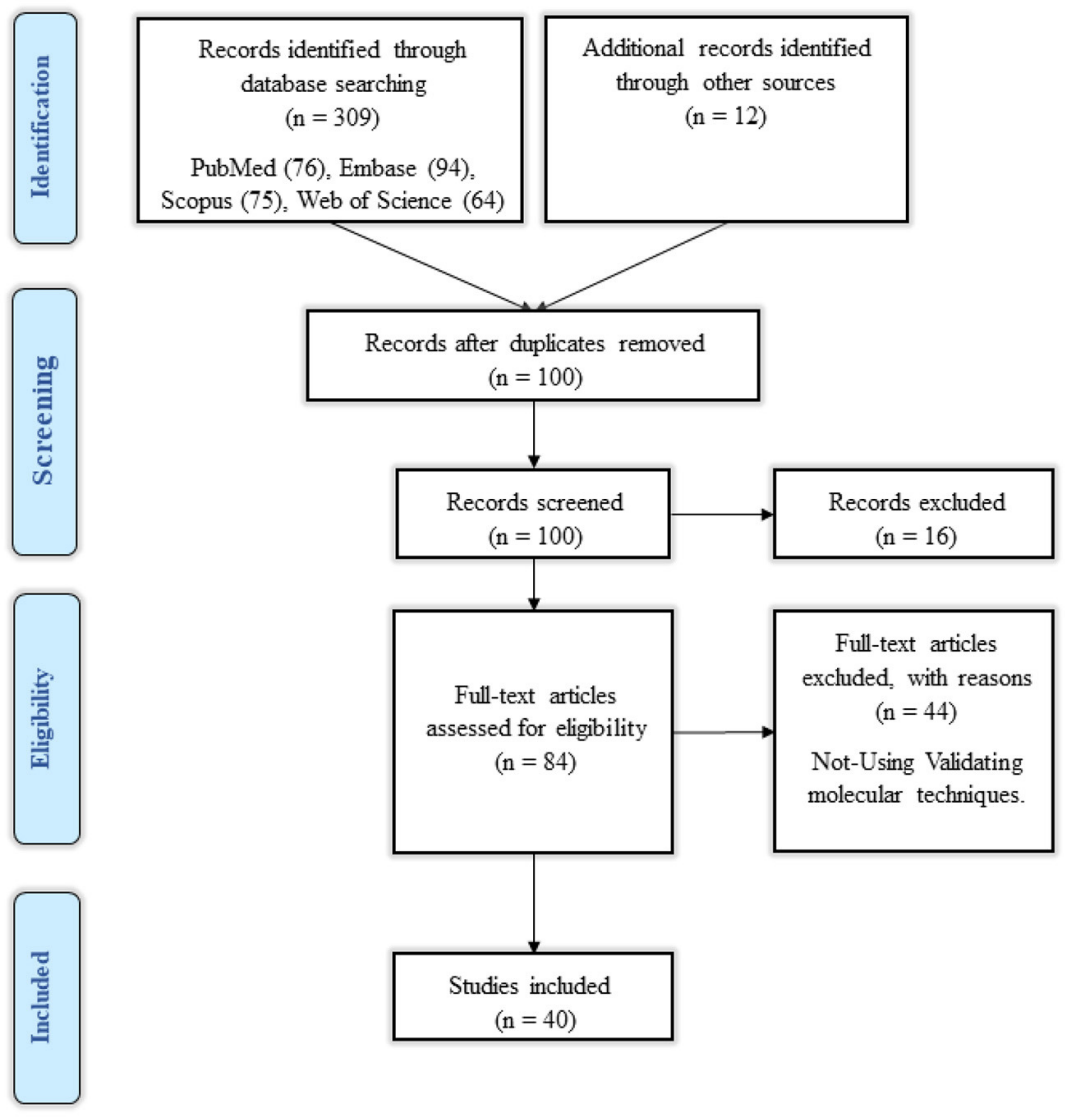
Flow chart of search strategy based on PRISMA flow diagram.

A total of 309 studies were discovered by searching the databases PubMed, Embase, Scopus, Web of Science, Google Scholar, ProQuest, and Cochrane, and 12 studies were identified by searching additional databases. After eliminating all duplicates, one hundred studies remained. The remaining articles' titles and abstracts were revised, and 16 studies were eliminated, leaving 86 studies. Each remaining article's full text was evaluated, and 46 studies were eliminated. Based on the given findings, the remaining 40 studies were all associated with our systematic review. The flowchart of the retrieval process utilized for the current study is shown in Figure 1.

**Figure 2:**
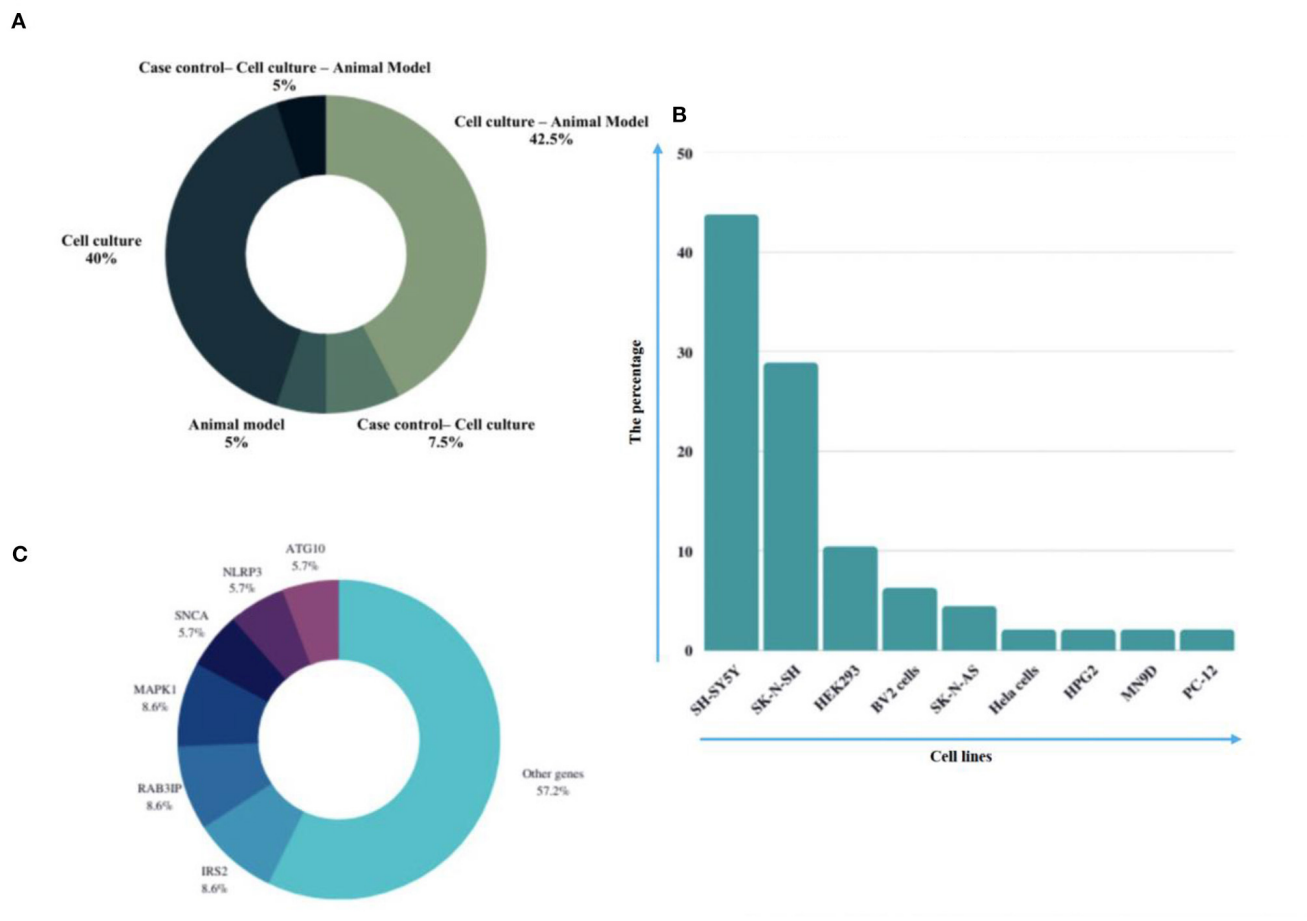
A summary of the qualified studies' details. **(A)** The design of studies. The majority of studies utilized cell culture and animal models (42.5%), and 40% of studies only utilized cell culture in their study design. **(B)** Percentage of cell lines utilized in the studies. SH-SY5Y (43.75%), SK-N-SH (22.88%), and HEK293 (10.48%) were the most used cell lines in the studies. **(C)** Percentage of targeted genes involved in ceRNA axes in PD. RAB3IP, SP1, and IRS2 were among the most targeted genes studied in PD's ceRNA axes. However, most of the axes involved novel genes, as provided by the other genes in the figure.

Eligible studies were published between 2017 and 2022, which are included in Table 1. Based on the declared number, these studies included 264 samples of PD patients and 195 healthy controls. The patients' and controls' gender is rarely mentioned. Two animal models (mice and *C. elegans*) were used in 21 studies; in the majority, mice were the only animals involved. SH-SY5Y, HEK293, Hela, HepG2, BV2 cells, SK-N-SH, SK-N-AS, MN9D, and PC-12 are the cell lines utilized in these studies. Figure 2 illustrates the frequency of the genes targeted in the studies. In particular, most studies are restricted to China, Italy, India, and Iran, each of which has one study on the ceRNA axes in PD.

**Table 1:** CeRNAs axes studies in PD.

**References**	**Origin**	**Type of study**	**Human samples**	**Cell line(s)**	**Animal model**	**CeRNA(s)**	**Shared miRNA(s)**	**Major methods**	**Major findings**
Liu et al. (2017)	China	Animal study Cell culture	**–**	SH-SY5Y HEK293	Mice (C57BL/6)	MALAT1 ↑ miR-124 targets	miR-124 ↓	qRT-PCR, western blot, TUNEL assay, flow cytometry, caspase3 activity analysis, luciferase assay	In mouse and *in vitro* models, MALAT1 induces apoptosis by sponging miR-124, which can probably be applied to treating PD MALAT1 knockdown improved miR-124 expression in MPTP/MPP+ initiated models of PD
Wu et al. (2017)	China	Animal study Cell culture	**–**	SH-SY5Y	Rat	ABCA1 ↓ A20	miR-873 ↑	IHC, qRT-PCR, immunoblotting, dual-luciferase assay, transient transfection, and luciferase assays	Inhibiting A20 could aggravate the NF-κB signaling pathway by up-regulating miR-873 miR-873 sponge might be able to reduce PD symptoms by up-regulating ABCA1 and A20 Inhibition of miR-873 may play a dual protective role in PD as it induces intracellular cholesterol homeostasis and improves neuroinflammation
Straniero et al. (2017)	Italy	Case-control Cell culture	Post-mortem Brains (51 cases and 42 controls)	Hela HEK293 HepG2	–	GBAP1 ↓ GBA	miR-22-3p ↑	qRT-PCR, luciferase assay, western blot, GCase enzyme activity assays, analysis of the splicing pattern, and iPSC derivation	During dopaminergic differentiation, GBA will increase, while miR-22-3p will decrease GBPA1 (pseudogene) functioned as a GBA ceRNA and was identified as the first microRNA controlling GBA Confirmation of GBA/GBAP1/miR22-3p dysregulation in PD patients may lead to new treatment strategies by modulating the NMD pathway to increase GBAP1 levels or directing miRNA/pseudogene expression
Cao et al. (2018)	China	Animal study Cell culture	**–**	BV2 cells	Mice (C57BL/6)	SNHG1 ↑ NLRP3	miR-7 ↓	Luciferase assay, RNA IP, IHC, qRT-PCR, western blot, ELISA, caspase-3 activity assay	Neuroinflammation is promoted by SNHG1 through the miR-7/NLRP3 pathway SNHG1 downregulation increases miR-7 expression and suppresses dopaminergic neuron loss, microglial activation, and NLRP3 inflammasome expression in midbrain SNpc in MPTP-treated mice SHNG1 may be a potential therapeutic target for PD
Kumar et al. (2018)	India	Animal study	**–**	–	*C. elegans* (N2 and NL5901 strain)	circzip-2 ↓ zip-2	miR-60 ↑	qRT-PCR, sanger sequencing, imaging of α-synuclein protein aggregation, RNA-seq, quantification of ROS, acetylcholine and acetylcholinesterase estimation using amplex red, lifespan assay	Circzip-2 may have a protective role against PD Losing circzip-2 enhances miR-60 activity, decreasing protective genes, and when ZIP-2 is silenced, the protective activity is restored through the daf-16 pathway
Sang et al. (2018)	China	Cell culture	**–**	SH-SY5Y	–	CircSNCA ↓ SNCA	MiR-7 ↑	qRT-PCR, western blot, luciferase assay, immunofluorescence localization, MTT assay	CircSNCA knockdown resulted in downregulation of alpha-synuclein (SNCA), which proved circSNCA inhibition was effective in PD treatment CircSNCA up-regulates SNCA through miR-7 sponging, which decreased apoptosis and endorsed cell autophagy in PD
Xu et al. (2018)	China	Animal models Cell culture	**–**	SH-SY5Y	Mice (C57BL/6)	lincRNA-p21 ↑ miR-1277-5p target	miR-1277-5p ↓	qRT-PCR, cell nucleus, and cytoplasm fraction isolation, western blot, RIP assay, dual-luciferase activity assay, cell viability assay, cell apoptosis assay	In SH-SY5Y cells treated with MPP+, the lincRNA-p21/miR-1277-5p axis modulated cell viability and apoptosis by targeting α-synuclein LincRNA-p21 may be a valuable target in the treatment of PD
Ding et al. (2019)	China	Cell culture	**–**	SH-SY5Y	–	linc-p21 ↑ TRPM2	miR-625 ↓	qRT-PCR, TUNEL assay, MTT assay, LDH assay, caspase-3 assay, measurement of ROS generation, measurement of SOD activity, western blot, luciferase assay	TRPM2 expression is positively regulated by lnc-p21 that interacts with miR-625, and knockdown of TRPM2 reduces MPP+-induced neuroinflammation The Lnc-p21-miR-625-TRPM2 regulatory axis was identified as a potentiating axis in the pathogenesis of PD
Lin et al. (2019)	China	Animal models Cell culture	**–**	SH-SY5Y	Mice (C57BL/6)	HOTAIR ↑ RAB3IP	miR-126-5p ↓	Apoptosis assay, luciferase assay, RIP assay, qRT-PCR, western blot, CCK-8 assay, flow cytometry, morris water maze test, pole test, IHC, TUNEL assay	HOTAIR promotes PD by influencing cell proliferation and apoptosis by sponging miR-126-5p and regulating RAB3IP Knockdown of HOTAIR decreased the number of α-synuclein-positive cells while reducing the apoptosis rate among dopaminergic neurons
Liu et al. (2020)	China	Cell culture	**–**	SK-N-SH	–	NEAT1 ↑ RAB3IP	miR-212-5p ↓	qRT-PCR, western blot, CCK8 assay, RIP assay, cell apoptosis assay, flow cytometry, LDH release assay, ROS activity assay, SOD activity assay, dual-luciferase assay	In MPP+-treated SKN-SH cells, NEAT1, and RAB3IP were increased, whereas miR-212-5p was downregulated NEAT1 knockdown reduced MPP+-induced apoptosis, inflammation, and cytotoxicity in SK-N-SH cells *via* up-regulating miR-212-5p
Xu et al. (2020)	China	Animal study Cell culture	**–**	BV2 cells	Mice (C57BL/6)	GAS5 ↑ NLRP3	miR-223-3p ↓	qRT-PCR, cell viability test, apoptosis test, dual-luciferase assay, western blot, IHC,	GAS5 positively regulates NLRP3 by competitive sponging of miR-223-3p GAS5 knocked down enhances the expression of miR-223-3p, which reduces inflammatory factors GAS5 promotes PD development by targeting the miR-223-3p/NLRP3 axis
Zhang L. et al. (2020)	China	Animal study Cell culture	**–**	SH-SY5Y	Mice (C57BL/6)	AL049437 ↑ MAPK1	miR-205-5p ↓	qRT-PCR, CCK-8 assay, flow cytometry, ELISA, ROS species assay, luciferase assay, RNA pull-down assay, western blot	By adjusting miR-205-5p/MAPK1 in SH-SY5Y cells, lncRNA AL049437 alleviates MPP+-induced neuronal injury A high level of AL049437 reduces the expression of miR-205-5p in SH-SY5Y cells
Zhang X. et al. (2020)	China	Animal study	**–**	–	Mice (C57BL/6J)	LOC102633466 ↑ LOC102637865 ↑ LOC102638670 ↑ Col6a1 and Wnt6	miR-505-5p ↓ miR-188-3p ↓ miR-873a-5p ↓	qRT-PCR, RNA-seq, FastQC, HE staining, bioinformatic analysis	LOC102633466, LOC102637865, and LOC102638670 were significantly up-regulated in the aerobic exercise training group compared with the PD group Aerobic exercise may improve PD by acting on these three lncRNAs with a ceRNA mechanism
Meng et al. (2021)	China	Cell culture	**–**	SK-N-SH	–	Linc00943 ↑ RAB3IP	miR-15b-5p ↓	qRT-PCR, Cell transfection, western blot, MTT assay, flow cytometry, ELISA, dual-luciferase reporter analysis, and RIP	The MPP+-stimulated SK-N-SH cells led to an increase in LINC00943 abundance Silencing LINC00943 prevented MPP+-induced cell viability loss and increased apoptosis, inflammatory damage, and oxidative injury LINC00943 suppressed miR-15b-5p, and inhibiting miR-15b-5p reversed LINC00943-mediated mitigation of MPP+-induced neuronal injury. RAB3IP is targeted by miR-15b-5p, and LINC00943 might control RAB3IP *via* miR-15b-5p
Zhao J. et al. (2020)	China	Animal study Cell culture	**–**	SH-SY5Y	Mice (C57BL/6)	SNHG1 ↑ PTEN	miR-153-3p ↓	qRT-PCR, western blot, MTT assay, flow cytometry, luciferase assay, RNA pull-down analysis, apoptosis assay, RIP assay	SNHG1 expression was increased in both MPTP-induced PD mice and MPP+-treated SH-SY5Y cells SNHG1 knockdown inhibited MPP+-induced cytotoxicity in SH-SY5Y cells through the miR-153-3p/PTEN pathway
Zhao J. Y. et al. (2020)	China	Cell culture	–	SK-N-SH	**–**	HOTAIR ↑ ATG10	MIR-874-5P ↓	qRT-PCR, Cell viability assay, flow cytometry, western blot, ELISA, measurement of LDH activity, detection of ROS generation, determination of SOD activity, dual-luciferase assay, RIP assay	Silencing of HOTAIR decreases MPP+-triggered neuronal damage by regulating ATG10, which inhibits the effect of miR-874-5p on it
Zhou et al. (2020a)	China	Cell culture	–	SK-N-SH SK-N-AS	**–**	NORAD ↓ SLC5A3	miR-204-5p ↑	qRT-PCR, cell viability assay, cell apoptosis assay, western blot, detection of lactate dehydrogenase level, assessment of oxidative stress, enzyme-linked immunosorbent assay, dual-luciferase assay, RNA immunoprecipitation	NORAD was able to up-regulate SLC5A3 with interacting miR-204-5p and protected neuroblastoma cells from MPP+-induced damage As a result, NORAD is a PD inhibitor
Zhou et al. (2020b)	China	Cell culture Animal study	–	SK-N-SH	Mice (C57BL/6)	SNHG14 ↑ KLF4	miR-214-3p ↓	qRT-PCR, cell viability and apoptosis assays, ELISA, bioinformatics and dual-luciferase assay, RIP assay, western blot	SNHG14 acts as a miR-214-3p sponge and up-regulates miR-214-3p to reduce damage to MPPCS-stimulated SK-N-SH cells by downregulating KLF4 In PD models, SNHG14 was up-regulated, and miR-2143-p was down-regulated
Feng et al. (2021)	China	Cell culture Animal study	–	HEK293	Mice (C57BL/6)	MiR-330 synthetic sponge SHIP1, NF-κB	MiR-330 ↓	Dual-luciferase assay, inhibitor and agonist of SHIP1, western blot, qRT-PCR, AGO2-immunoprecipitation, ELISA, immunofluorescence, flow cytometry	Treatment of the cells with miR-330 sponge suppresses LPS-induced chronic neuroinflammation in PD by down-regulating the activity of microglia with reduced inflammatory cytokines *via* the SHIP1/NF-κB signaling pathway
Lian et al. (2021)	China	Cell culture	–	SK-N-SH	–	LINC00943 ↑ CXCL12	miR-7-5p ↓	qRT-PCR, cell viability assay, cell apoptosis assay, western blot, measurement of ROS generation and SOD activity, dual-luciferase assay, RIP assay	LINC00943 acted as a miR-7-5p sponge and regulated CXCL12 expression in MPP+-induced SK-N-SH cells LINC00943 knockdown may partially reduce neuronal damage caused by MPP + due to that LINC00943 was increased in MPP+-inducted PD models
Liu et al. (2021)	China	Cell culture Animal study	–	SK-N-SH HEK293T	Mice (C57BL/6)	NEAT1 ↑ AXIN1	miR-212-3p ↓	qRT-PCR, CCK-8 assay, flow cytometry, western blot analysis, ELISA, dual-luciferase reporter assay, RIP assay, statistical analysis	Silencing of NEAT1 may hinder PD progression due to its up-regulation in MPTP-treated PD mouse models and MPP+-stimulated PD cell models MiR-212-3p regulated the cell progression by targeting AXIN1, which sponge with NEAT1
Shen Y. et al. (2021)	China	Cell culture Animal study	–	SH-SY5Y	BALB/c mice	MIAT ↑ SYT1	miR-34-5p ↓	FISH, dual-luciferase assay, RIP assay, qRT-PCR, western blot, CCK-8 assays, annexin V-fluorescein isothiocyanate (FITC)/propidium iodide (PI) dual label staining, IHC, TUNEL assay, behavioral assays	MIAT sponge miR-34-5p and then regulate the SYT1 expression, as a result, exerts neuroprotective effects in PD and promotes the expression of Parkin in the SH-SY5Y cells MIAT expression is downregulated in various brain regions and protects the neural function of SH-SY5Y cells
Shen Y. E. et al. (2021)	China	Cell culture Animal study	–	SH-SY5Y	Mice (C57BL/6)	PART1 ↓ MCL1	miR-106b-5p ↑	Western blot, qRT-PCR, CCK8 assay, apoptosis detection, caspase 3 activity detection, ELISA, ROS detection, LDH detection, SOD detection, dual-luciferase assay, RIP assay, RNA pull-down assays	PART1 is a protecting factor for PD by sponging miR-106b-5p, and MCL1 is a direct target for miR-106b-5p Consequently, PART1 reduced MPP+-induced damage to SHSY5Y cells Reduced expression of PART1 is shown in PD
Sun et al. (2021)	China	Cell culture	-	SH-SY5Y	-	NEAT1 ↑ GJB1	miR-1301-3p ↓	qRT-PCR, western blot analysis, cell apoptosis analysis, IL-1β production analysis, dual-luciferase activity assay	Knockdown of NEAT1 inhibits MPP+-Induced neuronal apoptosis in PD *via* inhibition of α-Syn-Induced activation of the inflammatory form NLRP3 through upgrading the expression of miR-1301-3p and downregulation of GJB1 NEAT1 acted as a therapeutic factor in PD
Wang S. et al. (2021)	China	Cell culture	–	SK-N-SH SK-N-AS	–	NEAT1 ↑ SP1	miR-519a-3p ↓	qRT-PCR, MTT assay, flow cytometry analysis, western blot, dual-luciferase assay	NEAT1 expression is significantly up-regulated in MPP+-induced SK-N-SH and SK-N-AS cells in PD Silencing of NEAT1 applied protective effects in MPP+-induced neuroblastoma cell injury *via* regulating miR-519a-3p, which reduces SP1 expression
Xiao et al. (2021)	China	Cell culture Animal study	–	SK-N-SH MN9D	Mice (C57BL/6)	SNHG1 ↑ MAPK1	miR-125b-5p ↓	qRT-PCR, CCK-8 assay, apoptosis assay, caspase-3 and caspase-9 activity assay, LDH release, ROS and SOD activity assay, ELISA, dual-luciferase assay, western blot	MiR-125b-5p is sponged by SNHG1, and it suppresses MAPK1 Silencing of SNHG1 and overexpression of miR-125b-5p applied neuroprotective actions in MPP+-evoked neuronal injury in SK-N-SH and MN9D Cells and MPTP-Induced PD mice
Xie et al. (2021)	China	Cell culture	–	SH-SY5Y	–	SOX21-AS1 ↑ IRS2	miR-7-5p ↓	qRT-PCR, cell viability assay, lactate dehydrogenase assay, TUNEL assay, flow cytometry apoptosis analysis, western blot, measurement of ROS generation, measurement of SOD activity, nuclear-cytoplasmic separation assay, fluorescent *in situ* hybridization assay, luciferase gene assay	SOX21-AS1 seems to tie with miR-7-5p, whose overexpression diminished MPP+- initiated cell damage IRS2 served as the target quality of miR-7-5p, and its expression was positively modulated by SOX21-AS1
Xu et al. (2021)	China	Cell culture	–	SH-SY5Y	–	ID2-AS1 ↑ IFNAR1	miR-199a-5p ↓	Lactate dehydrogenase assay, flow cytometry, reactive oxygen species activity, western blot, subcellular fraction analysis, RNA immunoprecipitation assay, ELISA, dual-luciferase assay	ID2-AS1 down-regulation weakened MPP+ induced cytotoxicity in SH-SY5Y cells Down-regulation of ID2-AS1 alleviated the neuronal damage in PD by regulating the miR-199a-5p/IFNAR1/JAK2/STAT1 axis
Zhang et al. (2021)	China	Case-control Animal model Cell culture	PD patients (36 cases and 20 controls)	SH-SY5Y	Rats	SNHG7 ↑ TRAF5 NF-κB	miR-425-5p ↓	Immunofluorescence, IHC, qRT-PCR, western blot, MTT assay, measurement of MDA, SOD, and GSH-PX, ELISA, RIP assay, luciferase assay	SNHG7 works as a ceRNA by sponging miR-425-5p and promoting TRAF5 Low expression of SNHG7 or miR-425-5p overexpression attenuates neuronal apoptosis
Chen C. et al. (2022)	China	Case-control Cell culture	PD patients (99 cases and 93 controls)	SH-SY5Y	–	RMST ↑ miR-150-5p targets	miR-150-5p ↓	qRT-PCR, flow cytometry, CCK-8 assay, luciferase reporter assay	Serum RMST is a potential biomarker for PD diagnosis Downregulation of RMST may influence the onset and progression of PD by suppressing neuron cell death and the production of inflammatory cytokines through miR-150-5p
Zheng et al. (2021)	China	Cell culture	–	SK-N-SH	–	UCA1 ↑ KCTD20	miR-423-5p ↓	qRT-PCR, cell viability and apoptosis assays, western blot, ELISA, dual-luciferase assay, RIP assay	MPP+ causes PD-like symptoms, and the silencing of UCA1 protects SK-N-SH cells from MPP+-evoked cytotoxicity by targeting the miR-423-5p/KCTD20 axis
Zhou Q. et al. (2021)	China	Cell culture Animal model	–	SH-SY5Y PC-12	Mice (C57BL/6)	XIST ↑ Sp1	miR-199a-3p ↓	CCK-8 assay, flow cytometry, cell apoptosis analysis by annexin-V/PI staining, TUNEL assay, TUBB3, luciferase assay, RIP analysis, TH detection by IHC, H&E and TUNEL staining, western blot, qRT-PCR	XIST sponges miR-199a-3p to modulate Sp1 expression Behavioral indications of PD were successfully lightened upon shXIST or miR-199a-3p treatment
Zhou S. F. et al. (2021)	China	Cell culture	–	SK-N-SH SH-SY5Y	–	NEAT1 ↑ ARHGAP26	miR-1277-5p ↓	qRT-PCR, MTT assay, flow cytometry, western blot, ELISA, measurement of MDA, LDH, SOD, and GSH-Px, dual-luciferase assay	NEAT1 knockdown and miR-1277-5p overexpression can mitigate MPP+-induced neuron injury Upregulation of NEAT1 might contribute to MPP+-induced neuron injury through the NEAT1-miR-1277-5p-ARHGAP26 axis
Chen C. et al. (2022)	China	Cell culture Animal model	–	SH-SY5Y	Mice (C57BL/6)	CircTLK1 ↑ DAPK1	miR-26a-5p ↓	Histological analysis, assessment of motor function, cell culture and treatment, western blot, qRT-PCR, LDH cytotoxicity assay, cell viability and apoptosis, dual-luciferase gene assay	circTLK1 expression increased in PD models. The knockdown of circTLK1 alleviated the PD-induced neuron injury. Overexpression of DAPK1 and inhibition of miR-16a-5p rescinded the protective function of shcircTLK1 in neurons
Sun X. M. et al. (2022)	China	Cell culture Animal model	–	SK-N-SH	Mice (C57BL/6)	LINC00943 ↑ SP1	miR-338-3p ↓	MTT assay, EdU staining, flow cytometry, western blot, ELISA assay, qRT-PCR, subcellular localization assay, dual-luciferase assay, RIP assay	LINC00943 works as a ceRNA by sponging miR-338-3p and positively regulating SP1 Knockdown of LINC00943 could alleviate nerve cell injury
Cao et al. (2022)	China	Cell culture	–	SH-SY5Y	–	circ_0070441 ↑ IRS2	miR-626 ↓	qRT-PCR, Western blot, ELISA, CCK-8, flow cytometry, caspase-3 assay, RIP	In MPP+-treated SH-SY5Y cells, circ_0070441, and IRS2 levels increased while miR-626 expression decreased. Circ_0070441 depletion reduced MPP+-induced neuronal damage *via* controlling cell death and inflammation. IRS2 was a miR-626 target, and Circ 0070441 behaved as a sponge for it
Sun Z. M. et al. (2022)	China	Cell culture	–	SH-SY5Y	–	SNHG10 ↑ IRS2	miR-1277-5p ↓	qRT-PCR, western blot, ELISA, CCK-8, apoptosis detection by flow cytometry, luciferase assay, RNA pull-down	SNHG10 knockdown abates MPP+-induced damage in SH-SY5Y cells through the miR-1277-5p/IRS2 axis Overexpression of IRS2 or inhibition of miR-1277-5p reverses the effect of SNHG10 knockdown
Zhuang et al. (2022)	China	Cell culture	–	SK-N-SH	–	SNHG14 ↑ ATG10	miR-519a-3p ↓	qRT-PCR, western blot, MTS cell viability assay, flow cytometry, dual-luciferase reporter assay, RIP	SNHG14 and ATG10 were both ceRNAs for miR-519a-3p, and ATG10 expression may be favorably regulated by SNHG14 by sponging miR-519a-3p Targeting SNHG14 and reinstating miR-519a-3p might protect DAn against MPP+ toxicity through ATG10 regulation
Zhang et al. (2022)	China	Case-control Animal model Cell culture	PD patient's blood (40 cases and 20 controls)	SH-SY5Y Microglia BV2	Mice (C57BL/6)	MIR17HG ↑ SNCA	miR-153-3p ↓	APO-induced rotation test, rotarod test, passive avoidance test, tissue immunofluorescence, IHC, qRT-PCR, Western blot, ELISA, detection of oxidative stress	MIR17HG is overexpressed in PD tissues and cells, and reducing MIR17HG expression reduces neuronal apoptosis and inflammatory responses in microglia in PD models and regulates PD progression by modulating the miR-153-3p/SNCA axis
Yousefi et al. (2022)	Iran	Cell culture Case-control	PD patients PBMC (38 cases and 20 controls)	HEK293T	–	linc0938 ↑ LRRK2 linc001128 ↓ ATP13A2	miR-24-3p ↓ miR-30c-5p ↓	Plasmid construction, Dual-luciferase reporter assay, qRT-PCR	linc001128, has-miR-24-3p, and miR-30c-5p expression were downregulated in PD patients Expression of Linc00938, LRRK2, and ATP13A2 were up-regulated in PBMC of the PD patients Linc00938 directly sponged miR-30c-5p in PD patients.

The upward arrow indicates the increased expression. The downward arrow indicates the decreased expression.

**Figure 3:**
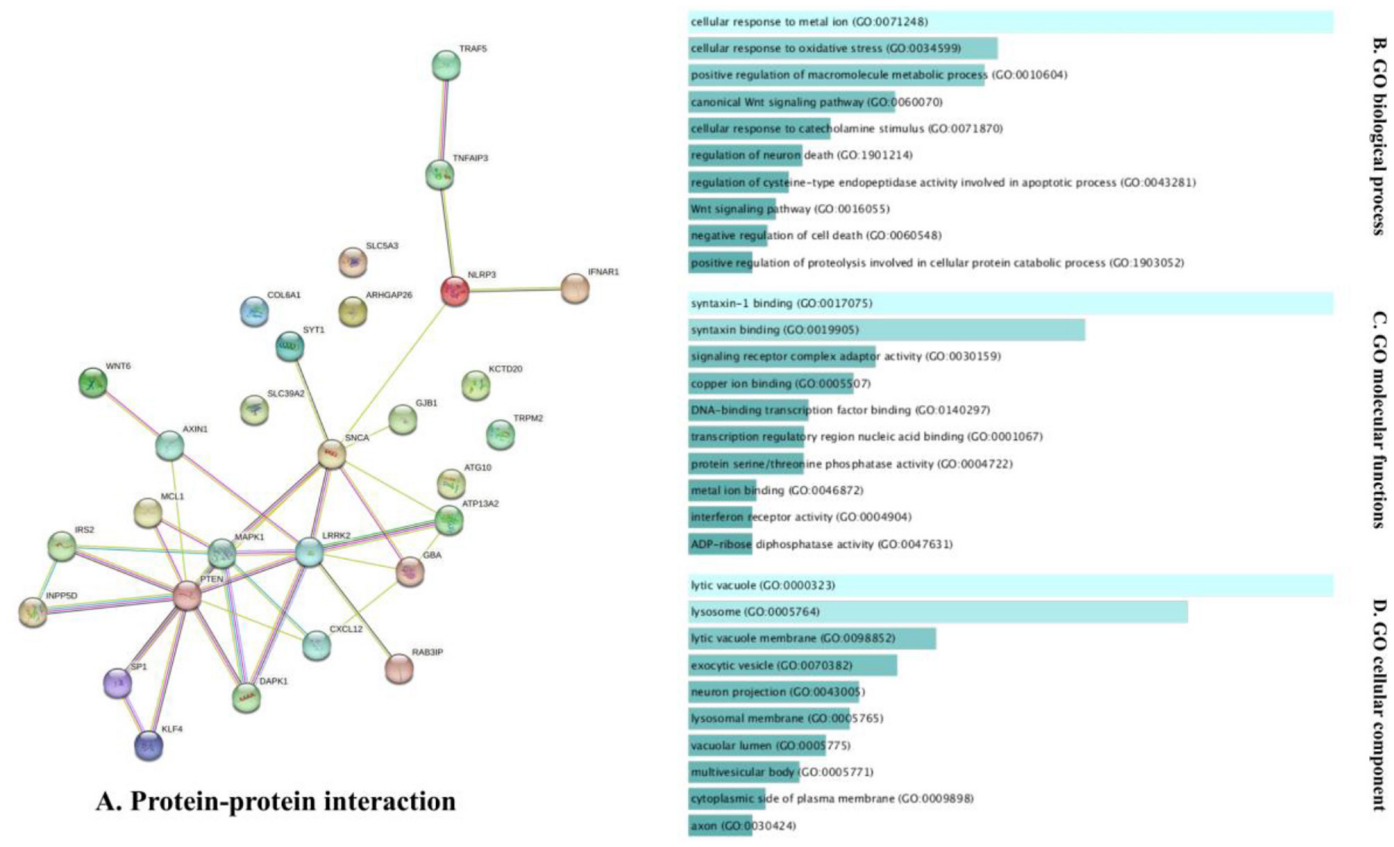
The PPI interaction and GO enrichment analysis of target genes involved in ceRNA axes in PD. **(A)** The PPI interaction was established using the STRING-DB and includes 29 nodes and 37 edges. **(B–D)** The GO enrichment of target genes on the verified ceRNA axes. The *P*-values are used to rank the importance of each group, with the length of each bar indicating the significance level. The order of the bars is based on the *p*-value. Note that the higher the association with a certain category, the less intense the color of the bars.

The analysis of the PPI network of the target genes of the ceRNA axes using STRING-DB identified a PPI network with 29 nodes and 37 edges, as demonstrated in Figure 3A. Furthermore, GO functional enrichment analysis indicated that shared genes were significantly enriched in numerous common biological processes, cellular compartments, and molecular functions of GO with *P* < 0.05 (Figures 3B–D). Among those significant pathways in biological processes, cellular response to metal ion, cellular response to oxidative stress, positive regulation of macromolecules metabolic process, canonical Wnt signaling pathway, and cellular response to catecholamine stimulus were among the processes with the highest number of genes. Cellular components were dedicated to lytic vacuole, lysosome, lytic vacuole membrane, and exocytic vesicle. Molecular functions were enriched in syntaxin-1 binding, syntaxin binding, signaling receptor complex adaptor activity, and copper ion binding. The expression levels of target genes in cell lines were provided in Supplementary Table 1 using the ProteomicsDB (Schmidt et al., 2018) based on the RNA-Seq method and transcripts per million (TPM) normalization method (Table 2).

**Table 2:** The expression levels of RAB3IP, IRS2, and MAPK1 in different cell lines.

**Gene symbol**	**Cell lines**	**Tissue synonym**	**Average TPM**	**Maximum TPM**	**Minimum TPM**
RAB3IP	CACO-2 cell	Unknown	55.9	55.9	55.9
	T-47D cell	Breast	44.8	44.8	44.8
	A-431 cell	Skin	37.8	37.8	37.8
	MCF-7 cell	Breast	35.9	35.9	35.9
	HaCaT cell	Unknown	28.7	28.7	28.7
	RPMI-8226 cell	Circulatory_system	24.9	24.9	24.9
	PC-3 cell	Prostate	23.4	23.4	23.4
	HEK-293 cell	Kidney	22.4	22.4	22.4
	Hep-G2 cell	Liver	21.9	21.9	21.9
	MOLT-4 cell	Circulatory_system	21.9	21.9	21.9
	HL-60 cell	Circulatory system	21.9	21.9	21.9
	HEL cell	Circulatory_system	21.6	21.6	21.6
	A-549 cell	Lung	21	21	21
	SK-BR-3 cell	Unknown	20.1	20.1	20.1
	U2-OS cell	Bone	19.1	19.1	19.1
	U-251 MG cell	Brain	15.8	15.8	15.8
	U-937 cell	Unknown	14	14	14
	K-562 cell	Circulatory_system	10.5	10.5	10.5
	SH-SY5Y cell	Autonomic_nervous_system	10.2	10.2	10.2
	THP-1 cell	Circulatory_system	7.5	7.5	7.5
	HeLa cell	Cervix_uterine	5.9	5.9	5.9
MAPK1	K-562 cell	Circulatory_system	287.7	287.7	287.7
	HL-60 cell	Circulatory system	87.2	87.2	87.2
	U2-OS cell	Bone	82.9	82.9	82.9
	HEL cell	Circulatory_system	69.3	69.3	69.3
	A-431 cell	Skin	67.5	67.5	67.5
	HaCaT cell	Unknown	63.4	63.4	63.4
	HEK-293 cell	Kidney	61.5	61.5	61.5
	MOLT-4 cell	Circulatory_system	57.7	57.7	57.7
	U-251 MG cell	Brain	44.5	44.5	44.5
	PC-3 cell	Prostate	43.4	43.4	43.4
	A-549 cell	Lung	43.4	43.4	43.4
	U-937 cell	Unknown	41.2	41.2	41.2
	SK-BR-3 cell	Unknown	40.3	40.3	40.3
	T-47D cell	Breast	38.2	38.2	38.2
	CACO-2 cell	Unknown	31.8	31.8	31.8
	Hep-G2 cell	Liver	30.9	30.9	30.9
	HeLa cell	Cervix_uterine	30.1	30.1	30.1
	SH-SY5Y cell	Autonomic_nervous_system	29	29	29
	THP-1 cell	Circulatory_system	24.6	24.6	24.6
	MCF-7 cell	Breast	24.3	24.3	24.3
	RPMI-8226 cell	Circulatory_system	8	8	8
IRS2	SH-SY5Y cell	Autonomic_nervous_system	42.8	42.8	42.8
	THP-1 cell	Circulatory_system	42.3	42.3	42.3
	HEK-293 cell	Kidney	27.1	27.1	27.1
	HL-60 cell	Circulatory system	8.5	8.5	8.5
	U-937 cell	Unknown	8.1	8.1	8.1
	U-251 MG cell	Brain	7.1	7.1	7.1
	HeLa cell	Cervix_uterine	6.3	6.3	6.3
	A-549 cell	Lung	5.9	5.9	5.9
	A-431 cell	Skin	5.1	5.1	5.1
	HEL cell	Circulatory_system	2.6	2.6	2.6
	HaCaT cell	Unknown	2.3	2.3	2.3
	MCF-7 cell	Breast	2.3	2.3	2.3
	RPMI-8226 cell	Circulatory_system	1.9	1.9	1.9
	K-562 cell	Circulatory_system	1.7	1.7	1.7
	PC-3 cell	Prostate	1.3	1.3	1.3
	T-47D cell	Breast	0.8	0.8	0.8
	CACO-2 cell	Unknown	0.5	0.5	0.5
	MOLT-4 cell	Circulatory_system	0.1	0.1	0.1
	SK-BR-3 cell	Unknown	0.1	0.1	0.1

## 4. Discussion

### 4.1. ncRNAs mechanism of actions

In particular, the ncRNAs that were involved in the ceRNA network in this study included miRNAs, lncRNAs, circRNAs, and pseudogenes, each of which exerts its effect through specific mechanisms; One of these mechanisms is sponging the target miRNA, which is involved in ceRNA axes (Moreno-García et al., 2020). Most research has shown that miRNA binds to specific regions of the 3′ UTR of its target mRNA, causing translation suppression, as well as decomposition of the mRNA adenylation and capping structure (Ipsaro and Joshua-Tor, 2015). Other mRNA regions containing miRNA binding sites have been discovered, including the 5′ UTR and the coding sequence, as well as inside the promoter areas (Xu et al., 2014). MiRNAs bound to the 5′ UTR and coding regions were found to suppress gene expression (Forman et al., 2008; Zhang J. et al., 2018), while miRNAs bound to gene promoters were found to stimulate transcription (Dharap et al., 2013). Furthermore, lncRNAs can exert their influence as guides, signals, decoys, and scaffolds in gene expression regulation (Wang and Chang, 2011). As a guide, lncRNAs can be incorporated into proteins such as chromatin-modifying enzymes, leading them to specific targets, thus mediating epigenetic change, and may modify the direction of gene expression in cis or trans through this advance. For example, ANRIL, XIST, HOTAIR, and KCNQ1OT1 lncRNAs can be used as chromatin modification enzymes that changes the epigenetic state (Bhat et al., 2016). LncRNA can also be used as a molecular signal, changing the structure of the chromatin, and recruiting transcription factors into the target gene, thereby increasing gene expression (Wang and Chang, 2011; Bhat et al., 2016). The functional versatility of lncRNA as a decoy mechanism allows it to behave as a “molecular sink” of RNA binding proteins (RBPs), which these groups of lncRNAs may be negative modulators, including transcription factors, regulatory factors, and chromatin modulators (Balas and Johnson, 2018). One of the advantages of LncRNAs is that they act as scaffolds to attach multiple efficient molecular partners with the capability of triggering or suppressing transcription and transfer them simultaneously to specific locations during transcription (Wang and Chang, 2011; Bhat et al., 2016).

In addition, it has been discovered that circRNAs have many possible biological activities depending on their properties. Nuclear persistent circRNAs have the ability to influence transcription and splicing (Prats et al., 2020). CircRNAs may perform their functions *via* associating with proteins, such as functioning as a protein sponge, protein scaffolding, and protein recruiting (Huang et al., 2020). In particular, circRNAs are capable of being translated, However, due to this ability, it is possible that circRNAs are not considered as ncRNAs, which requires potential studies (Miao et al., 2021). On the other hand, pseudogenes can affect gene expression (not just the parent gene) at the transcription and post-transcription levels (Qi et al., 2015). A pseudogene may engage with a gene promoter at the transcriptional level. For example, pseudogene-generated antisense RNA interacts with mRNA with the same parent gene strand and blocks translation, and contributes to the creation of siRNA that can suppress the parent gene's expression (Hu et al., 2018).

### 4.2. ceRNAs axes potentials, from concepts to actual application

The ceRNA profile in PD may be different from the normal condition since the PD transcriptome differs significantly from the normal equivalents (Kurvits et al., 2021). Notably, ceRNA network regulation may be complicated. Predictions from the TargetScan database show that half of all miRNAs target 1–400 mRNAs and that a small percentage of miRNAs potentially target > 1,000 mRNAs. TargetScan also predicts that most ceRNAs include 1–10 MREs (Ala et al., 2013). As a result, multiple ceRNA-miRNA interactions result in very complex ceRNA networks (ceRNETs). It should be noted that studies in the field of ceRNA axes of PD are experiencing an upward trend, and since miRNAs are at the center of the ceRNA axes, it is fair to consider ceRNA related to PD as therapeutic targets, considering that miRNAs were introduced as a beneficial target in the measures of therapeutics of PD (Alieva et al., 2015; Nies et al., 2021). Feng et al. for example, used a synthetic sponge for miR-330 and suppressed miR-330, reducing chronic neuroinflammation in PD by decreasing inflammatory cytokines through the SHIP1/NF-B signaling pathway (Feng et al., 2021). In this regard, Titze-de-Almeida et al. thoroughly analyzed miRNAs as possible treatment targets for PD, analyzing their involvement in the disease's underlying processes, the approaches for suppressing aberrant expressions, and the existing technology for converting these small molecules from the laboratory to the clinic (Titze-de-Almeida et al., 2020).

In addition, the bioinformatic results of the current study, based on the enrichments of target genes involved in verified ceRNA axes, demonstrated that these genes are involved in critical pathways in PD. The cellular response to metal ion was the process in which most of the genes were enriched. It has been postulated that the toxicity of several peptides that aggregate may be connected to their capacity to attach ions of transition metals (Castellani et al., 2000). The involvement of metal ions in the pathogenesis of PD was reviewed in several studies (Castellani et al., 2000; Wei et al., 2021). Tosato and Di Marco also studied metal chelating treatment in PD, hypothesizing that around 250 metal-chelating characteristics toward Cu(II), Cu(I), Fe(III), Fe(II), Mn(II), and Zn(II) may be implicated in metal dyshomeostasis during PD (Tosato and Di Marco, 2019). Importantly, Deas et al. highlighted that α-synuclein oligomers' interactions with metal ions might lead to oxidative stress in neurons produced from human iPSCs (Deas et al., 2016). Another notable process in the GO enrichment of the targeted genes implicated in the ceRNA axis in PD was the cellular response to oxidative stress. Evidence suggests that oxidative damage and mitochondrial malfunction contribute to the chain of events that lead to dopaminergic neurodegeneration (Beal, 2005; Jenner and Olanow, 2006; Parker et al., 2008; Winklhofer and Haass, 2010; Schapira and Jenner, 2011). It should be emphasized that dopaminergic neuron loss is a crucial factor in PD development (Mamelak, 2018). Remarkably, the results of the bioinformatics study with enrichment in the pathways that play a vital role in the development of PD emphasize the importance of these ceRNA axes in the pathogenesis of PD. On the contrary, the bioinformatics part of this study only focused on the enrichment of genes targeted in the ceRNA axes. And future bioinformatics research may explore this subject more thoroughly to highlight these axes' significance.

However, various challenges must first be overcome in order to bring the notion to fulfillment. At first, it is better to select axes that play a vital role, are known as hubs, and are connected to many post-transcriptional regulatory components that cannot be supplanted by the cell. One of the topics to consider in the following is the preceding one's emphasis; it seems almost impossible to impact an axis without altering ceRNET. Non-specific modification of the ceRNA network is dangerous because it has the ability to alter regular gene expression in unexpected ways. Finally, more studies are required to create therapeutic techniques and delivery systems for ceRNAs. However, vectors developed for gene therapy studies have the potential to be widely used in ceRNA-related research.

### 4.3. NEAT1-associated ceRNA axes in PD

**Figure 4:**
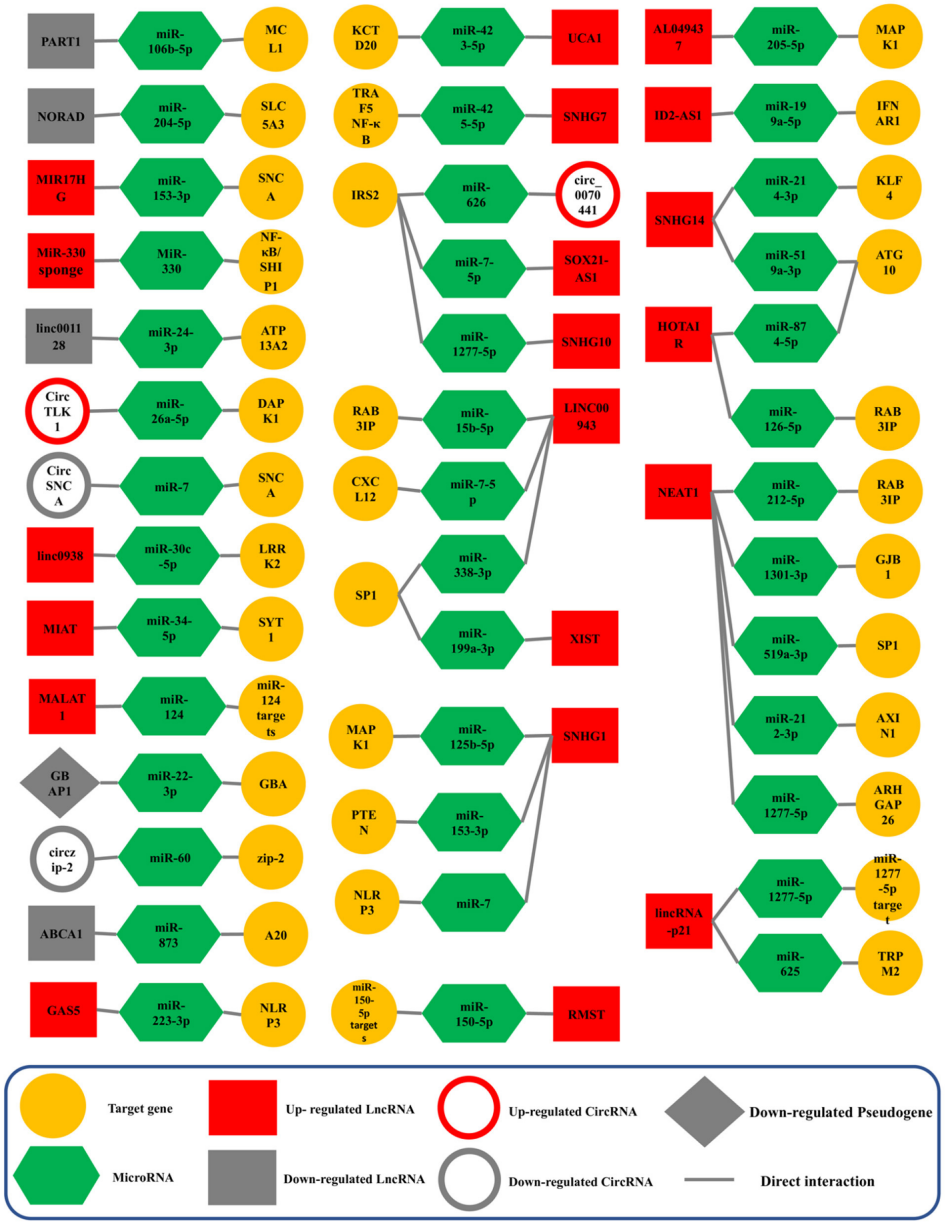
Validated ceRNA axes in PD. The red and gray colors of the squares indicate an increase and a decrease in the expression of lncRNAs, circRNAs, and pseudogenes, respectively. This schematic figure shows the importance of the constituent components of ceRNA axes. In addition, this figure shows that the studies around which of these components have been more extensive. NEAT1, the SNHG family, and LINC00943 were among the lncRNAs studied most on these axes. CircRNA, circular RNA; lncRNA, long non-coding RNA; miRNA, microRNA.

NEAT1 (Nuclear Enriched Abundant Transcript 1) lncRNA was first described in 2007 and later altered to Nuclear Paraspeckle Assembly Transcript (Hirose et al., 2014). In humans, NEAT1 is transcribed from the multiple endocrine neoplasia type I (MEN 1) gene that is located on chromosome 11 (Yu et al., 2017). There are two isoforms for NEAT1 transcription; the shorter NEAT1 (NEAT1S) is 3,684 nucleotides, while the longer NEAT1 (NEAT1L) is 22,743 nucleotides (Boros et al., 2021). Studies in humans and other models have shown that NEAT1 may participate as a critical factor in neurodegenerative diseases, human tumors, and cancers. It has the potential as a biomarker and as a therapeutic target for PD (Asadi et al., 2021; Boros et al., 2021). In PD, NEAT1 sponging five different ceRNA axes, through NEAT1/miR-212-3p (Liu et al., 2020), NEAT1/miR-1301-3p (Sun et al., 2021), NEAT1/miR-519a-3p (Wang S. et al., 2021), NEAT1/miR-212-5p (Liu et al., 2021), NEAT1/miR-1277-5p (Zhou S. F. et al., 2021; Figure 4).

MiR-212-3p regulated the cell's progression by targeting AXIN1 (axis inhibition protein 1), which provides an interacting target for NEAT1. AXIN1 is a multifunctional scaffold protein whose functional role is related to the progression of many diseases (Li et al., 2013). Many current studies have shown that AXIN1 can act as a tumor suppressor to participate in the tumorigenesis (Saeed, 2018). Saeed identified that AXIN1 could serve as a new gene for PD through a GWAS meta-analysis (Saeed, 2018). Liu et al. found that NEAT1 was overexpressed in PD and that upregulation of AXIN1 resulted from the inhibition of miR-212-3p. As a result, the silencing of NEAT1 by affecting miR-212-3p/AXIN1 may hinder PD progression (Liu et al., 2021). Moreover, in the NEAT1/miR-1301-3p axis, GJB1 (Gap Junction Protein Beta 1), which encodes a member of the gap junction protein family, was regulated. Gap junction proteins are channels that facilitate the movement of ions and small molecules between cells (Zhang Z. et al., 2020). Recently, Reyes et al. have demonstrated that GJB1 is involved in the preferential uptake of α-syn oligomeric in neurons and that the upregulation of GJB1 is associated with α-syn accumulation in a PD model. This study indicated the potential and crucial role of GJB1 in the α-syn-induced neuronal apoptosis (Reyes et al., 2019). Sun et al. revealed that overexpression of NEAT1 was seen in PD while miR-1301-3p was down-regulated, and subsequent increased GJB1 and knockdown of NEAT1 may hinder PD progression (Sun et al., 2021). In addition, Wang et al. revealed that NEAT1 expression was significantly up-regulated in the NEAT1/miR-519a-3p axis in PD. In this axis, SP1 is overexpressed by down-regulation of miR-519a-3p (Wang S. et al., 2021). This gene produces a zinc-finger transcription factor that can activate or suppress transcription in response to stimuli. It binds to GC-rich motifs with high affinity and controls many gene expressions involved in various processes (Koutsodontis et al., 2002). Research revealed that SP1 might ameliorate PD-linked neuropathology by regulating leucine-rich repeat kinase 2 (LRRK2) transcription (Wang and Song, 2016). In addition, Chen et al. demonstrated that SP1 knockdown hindered the inhibitory impact of MPP+ exposure on endothelial protein C receptor (EPCR) in PD (Chen et al., 2015).

On the other axis, NEAT1/miR-212-5p, NEAT1 sponged miR-212-5p, and regulated RAB3A Interacting Protein (RAB3IP) expression (Liu et al., 2020). RAB3IP, a significant activator of Rab proteins, has been shown to affect neurite outgrowth and PD progression (Homma and Fukuda, 2016; Lin et al., 2019). Liu et al. reported that this lncRNA and RAB3IP were increased, whereas miR-212-5p was down-regulated in PD. Therefore, NEAT1 knockdown may play a protective role in PD (Liu et al., 2020). Likewise, NEAT1 regulated miR-1277-5p expression and targeted Rho GTPase Activating Protein 26 (ARHGAP26) (Zhou S. F. et al., 2021). Cell-extracellular matrix interactions initiate a signaling cascade that triggers integrin cell surface receptors and regulates actin cytoskeleton organization. One of the proteins implicated in these cascades is focal adhesion kinase, a GTPase-activating protein encoded by this gene that binds to focal adhesion kinase and intervenes in the activity of the GTP-binding proteins RhoA and Cdc42 (Lopez-Ilasaca, 1998). In this respect, Zhou et al. discovered that the upregulation of NEAT1 might contribute to MPP+-induced neuron injury through the NEAT1-miR-1277-5p-ARHGAP26 axis in PD. Hence, NEAT1 knockdown and miR-1277-5p overexpression can mitigate neuron injury (Zhou S. F. et al., 2021). Studies analyzing NEAT1 have revealed the significance of this lncRNA in PD due to its potential role in ceRNA expression axes and have certainly made it a challenging target.

### 4.4. SNHG1, SNHG7, SNHG10, and SNHG14-associated ceRNA axes in PD

Small molecule RNA host gene 1 (SNHG1), also known as linc00057, is a lncRNA that was recently found and transcribed from UHG and is present on the 11q12.3 chromosome (Zhao J. et al., 2020). It was observed that SNHG1 was up-regulated in diverse types of cancer and that it served as a prognostic factor (Zhao et al., 2018). SNHG1 is also proven to elevate inflammation and autophagy of neurons in PD (Sabaie et al., 2021). The participation of SNHG1 in PD has been studied along several ceRNA axes, including SNHG1/miR-7/NLRP3, SNHG1/miR-125b-5p/MAPK1, SNHG1/miR-153-3p/PTEN. It should be noted that SNHG1 experiences up-regulation in all three axes (Cao et al., 2018; Zhao J. et al., 2020). Nod-like receptor protein 3 (NLRP3) activation results in the activation of caspase-1, cleaving interleukin (IL)-1β, and IL-18 into their active forms in response to infectious agents and cellular damage (Cao et al., 2018; Kelley et al., 2019). Cao et al. revealed that neuroinflammation is promoted by SNHG1 through the miR-7/NLRP3 pathway (Cao et al., 2018). MAPK1, a serine/threonine kinase member of the MAPK family, participates in several biological functions, including the cell cycle, cell death, and cell survival (Dzamko et al., 2014). Previous research has shown a link between MAPK1 and the progression of PD (Triplett et al., 2015). MAPK has been identified as a potent activator of neuronal apoptosis, and its overactivation results in neuronal death in PD (Mielke and Herdegen, 2000; Choi et al., 2014). Xiao et al. revealed that SNHG1 sponges miR-125b-5p and that it suppresses MAPK1 (Xiao et al., 2021). Phosphatase and tensin homolog (PTEN) is a kind of tumor suppressor gene that adjusts cell growth and cell death in cancers and neurodegenerative diseases (Ismail et al., 2012). A potential therapeutic target for the neurodegenerative disease could be PTEN, which could function as a moderator of Reactive Oxygen Species (ROS) generation in the neuronal death (Zhao J. et al., 2020). Zhao et al. revealed that the PTEN/AKT/mTOR signaling pathway would be activated in SH-SY5Y cells by targeting miR-153-3p (Zhao J. et al., 2020).

SNHG7 is an oncogenic lncRNA on chromosome 9q34.2 and is highly expressed in cancer cells and tumors (Bian et al., 2020). Tumor necrosis factor receptor-associated factor 5 (TRAF5) is a cytoplasmic adapter that can trigger the NF-κB signaling pathway *via* its receptors and can take part in modulating nerve cell death, inflammatory reactions, and other neurological processes (Zhang et al., 2021). Zhang et al. demonstrated that in PD, SNHG7 works as a ceRNA by sponging miR-425-5p and promoting TRAF5 (Zhang et al., 2021). In addition, SNHG10 is a lncRNA located on chromosome 14q32.13 and is involved in advancing several types of cancer (Sun Z. M. et al., 2022). Insulin substrate receptor 2 (IRS2) is a cytoplasmic adaptor protein of the insulin signaling pathway and is a target of miR-1277-5p and shows a high expression level in the PD model. Sun et al. found that the knockdown of SNHG10 attenuates MPP+-induced damage in SH-SY5Y cells *via* the miR-1277-5p/IRS2 axis in PD (Sun Z. M. et al., 2022).

SNHG14 is detected on chromosome 15q11.2 and functions as an oncogene in cancers, kidney injury, and neurodegenerative diseases such as PD and AD, working as ceRNA (Qi et al., 2017; Wang et al., 2018; Zhuang et al., 2022). It was proved that SNHG14 is up-regulated in two ceRNA regulatory axes, including SNHG14/miR-519a-3p/ATG10 and SNHG14/miR-214-3p/KLF4 (Zhou et al., 2020b; Zhuang et al., 2022). Autophagy-related 10 (ATG10) is an E2-like enzyme involved in the autophagy (Zhang M.-Q. et al., 2018). Zhuang et al. revealed that the expression of ATG10 is positively regulated by SNHG14 through sponging miR-519a-3p (Zhuang et al., 2022). On the other hand, Krüppel-like factor 4 (KLF4) suppresses the cell cycle and is a kind of transcription factor, and it was shown that it could be pathogenic in AD and several human diseases (Zhou et al., 2020b). Zhou et al. disclosed that SNHG14, acting as a miR-214-3p sponge and up-regulating miR-214-3p, reduced damage to MPPCS-stimulated SK-N-SH cells by downregulating KLF4 (Zhou et al., 2020b). Altogether, studies have shown us that these members of the SNHG family can have a potential role as ceRNA in PD and can be promising in finding therapeutic approaches.

### 4.5. HOTAIR, MALAT1, and XIST associated ceRNA axes in PD

HOX antisense intergenic RNA (HOTAIR) is a 2.2 kb antisense transcript from the Homeobox C (HOXC) gene cluster located on 12q13.13 (Zhu and Zhu, 2022). HOTAIR has been shown to affect PD progression, although the exact function of this lncRNA remains unknown. In this respect, Lin et al. found that lncRNA HOTAIR is overexpressed in PD and influences the expression levels of RAB3IP by sponging miR-126-5p. The study shows that HOTAIR Knockdown has reduced the number of α-synuclein-positive cells (Lin et al., 2019). Similarly, Zhao et al. revealed that HOTAIR also experienced an up-regulation and regulated ATG10 expression by sponging miR-874-5p (Zhao J. Y. et al., 2020). The potential role of lncRNA HOTAIR makes it a challenging target for neurodegenerative diseases such as PD.

The metastasis-associated lung adenocarcinoma transcript 1 (MALAT1) lncRNA is located on human chromosome 11q13 (Zhang et al., 2017). Alternative splicing, transcriptional control, post-transcriptional control, and miRNA sponge interactions are just a few of the pathways in which lncRNA is implicated (Shi et al., 2015). MALAT1, which is abundantly expressed in brain tissues, is likely involved in forming synapses and other neurophysiological pathways (Abrishamdar et al., 2022). In this regard, Liu et al. reported that PD-related cell apoptosis is facilitated by MALAT1 through sponging miR-124. It is comprehended that MALAT1 is overexpressed in the PD model; however, additional research is necessary (Liu et al., 2017). X-inactive specific transcript (XIST) lncRNA is a crucial cell growth and development regulator. In addition to its original role in X-chromosome dosage adjustment, XIST acts as a ceRNA and contributes to the growth of tumors and other human disorders (Wang et al., 2021). Remarkably, Zhou et al. revealed that to regulate Sp1 expression, XIST sponges miR-199a-3p (Zhou Q. et al., 2021). Treatment with shXIST or miR-199a-3p successfully reduced behavioral signs of PD (Zhou Q. et al., 2021). Due to its crucial function in X-chromosome inactivation, XIST is a prominent lncRNA; nonetheless, more research is required to fully understand its function in PD.

### 4.6. linc-p21, LINC00943, LINC0938, and LINC001128 associated ceRNA axes in PD

Long intergenic non-coding RNAs (lincRNAs) are ncRNAs that are autonomously produced, have a length of more than 200 nucleotides, and do not overlap with identified coding genes. LincRNAs share characteristics with other members of the lncRNA family and account for more than half of all lncRNA transcripts in humans. LincRNAs were initially proposed by studies utilizing tiling arrays spanning genomic sequences, which discovered widespread transcription (Venter et al., 2001; Djebali et al., 2012) from areas with no identified coding genes (Kapranov et al., 2002; Rinn et al., 2003; Bertone et al., 2004; Maeda et al., 2006). The analysis of chromatin state signatures in murine cell types gave early evidence for the existence of functioning transcription units at the potential loci of these transcripts (Salditt-Georgieff and Darnell, 1982; Guttman et al., 2009). Because many lincRNAs overlap sequences with coding loci, they have been differentiated from the larger lncRNA class of transcripts. However, many publications fail to distinguish between these two types of transcripts and bundle them together as “lncRNAs.” The rapid discovery and annotation of intergenic and genic lncRNAs have led to a growing understanding of the non-coding RNA's roles (Salditt-Georgieff and Darnell, 1982; Carninci et al., 2005).

The long intergenic non-coding RNA p21 (linc-p21) is localized on chromosome 6p21 (Amirinejad et al., 2020). It was initially discovered by stimulating the mouse's p53-dependent apoptosis (Ding et al., 2019). According to Ding et al. the linc-p21/miR-625/TRPM2 axis has a role in the etiology of PD. TRPM2 expression is increased by linc-p21, which interacts with miR-625, and TRPM2 knockdown lowers the MPP+-induced neuroinflammation (Ding et al., 2019). Similarly, Xu et al. discovered that linc-p21 sponges, miR-1277-5p, and the axis altered cell survival and apoptosis in MPP+-treated SH-SY5Y cells (Xu et al., 2018). linc-p21 is overexpressed in both the former and latter axes, and the probable involvement of linc-p21 in PD requires further investigation.

Long intergenic non-protein-coding RNA 943 (LINC00943) is a recently identified epigenetic transcript shown to be aberrantly produced in PD (Zhou et al., 2018). Furthermore, it was found that the downregulation of LINC00943 had a useful function in decreasing MPP+-induced neurotoxicity in SK-N-SH cells in an *in vitro* cellular model of PD (Meng et al., 2021). LINC00943 suppresses miR-15b-5p while increasing RAB3IP expression in PD (Meng et al., 2021). According to Lian et al. LINC00943 worked as a miR-7-5p sponge and controlled CXCL12 expression in MPP+-induced SK-N-SH cells, and LINC00943 knockdown may partly alleviate neuronal damage caused by MPP+, as LINC00943 was raised in MPP+-induced PD models (Lian et al., 2021). Similarly, Sun et al. demonstrated that knocking down LINC00943 might reduce nerve cell damage in a model of PD animal model. Linc00943 may favorably regulate specificity protein 1 (SP1) by interacting with the ceRNA axis and inhibiting miR-338-3p (Sun X. M. et al., 2022). SP1 is a C2H2 zinc finger-structured DNA-binding protein that regulates gene transcription in some physiological and pathological processes (Chu, 2012). Monoamine oxidase B (MAO B) inhibitors, which block dopamine breakdown by inhibiting MAO B activity, are licensed and extensively used in the therapeutic treatment of PD (Yao et al., 2018). The promoter of the MAO B gene includes SP1 binding regions and its expression is directly regulated by SP1 (Shih et al., 2011).

Furthermore, Yousefi et al. investigated LINC0938 and LINC001128 involved in PD (Yousefi et al., 2022). Notably, LNC0938 was up-regulated, and on the other hand, LINC001128 was down-regulated. These two lincRNAs could be involved in ceRNA axes, including LINC0938/miR-24-3p/LRRK2, and LINC00112/miR-30c-5p/ATP13A2. Linc00938 could bind directly to hsa-miR-30c-5p, thus possibly regulating LRRK2 expression through the miR-30c-5p sponge (Yousefi et al., 2022). LRRK2 has a role in mitochondrial malfunction, intracellular ATP total, mitochondrial fission, mitochondrial transport, and oxidative stress (Sai et al., 2012; Park et al., 2018). Patients with the LRRK2 G2019S homozygous or R1441C heterozygous mutation have higher mitochondrial DNA (mtDNA) damage (Sanders et al., 2014). Another study found that LRRK2 is required to avoid ER stress and spontaneous neurodegeneration in *C. elegans* models missing the LRRK2 homolog (Sämann et al., 2009). On the other hand, on another axis, the increase in the ATP13A2 expression could be justified by two independent methods, one by binding hsa-miR-24-3p to ATP13A2 and the other by directly binding Linc01128 to ATP13A2. Therefore, the hsa-miR-24-3p and Linc01128 levels were decreased in PD; as a result, the ATP13A2 expression was increased in this disease (Yousefi et al., 2022). In this regard, the studies on the ceRNA axes involving lincRNAs must be continued in PD.

### 4.7. CircRNAs-associated ceRNA axes in PD

RNA sequencing is presently the only method that can provide a comprehensive landscape of circRNAs across the body and, in particular tissue locations (Philips et al., 2020). Specifically, ribo-minus RNA-Seq has enabled the identification of novel changes in circRNA expression as well as the investigation of these circRNAs' involvement in the condition of concern (Cooper et al., 2018). Circular RNAs are exceptionally stable regulatory molecules inside the cell because they are immune to exonuclease actions (Xie et al., 2017). CircRNAs have been characterized as protein decoys, scaffolds, and recruiters, as well as transcriptional regulators, miRNA sponges, and protein templates (Zhou W. Y. et al., 2020; Asadi et al., 2022). CircRNAs have an active role in muscular tissue formation (Legnini et al., 2017), synapse formation and activity field (Chen et al., 2019), neuronal gene expression regulation (van Rossum et al., 2016), and CNS differentiation and development (Mehta et al., 2020). CircRNAs are found in many different cell types but are especially abundant in neurons (Legnini et al., 2017; D'Ambra et al., 2019; Zhou W. Y. et al., 2020).

Notably, investigations into the involvement of circRNAs in PD are rising, and the roles of various circRNAs in the ceRNA axis have been established. In this regard, a circRNA derived from the SNCA gene (has_circ 0127305, commonly known as circSNCA) functions as a ceRNA of miR-7, up-regulating SNCA in PD. Moreover, pramipexol (PPX), a PD medication, suppresses circSNCA expression. Accordingly, Sang et al. demonstrated that inhibiting circSNCA and SNCA reduces apoptosis and promotes autophagy, reducing the development of PD (Sang et al., 2018). Furthermore, Chens et al. revealed increased circTLK1 expression in PD models (Chen W. et al., 2022). CircRNA circTLK1, encoded from TLK1 mRNA, was shown to be an oncogene in a renal cell cancer investigation. CircTLK1 acts as a molecular sponge for miR-136-5p, which increases CBX4 expression and promotes the development of renal cell carcinoma (Li et al., 2020). Afterward, Wu et al. discovered that circTLK1 had pro-ischemic stroke effects (Wu et al., 2019). Song et al. revealed the role of circTLK1 in myocardial ischemia/reperfusion damage (Song et al., 2020). The elimination of circTLK1 reduced PD-induced neuron damage. Overexpression of DAPK1 and suppression of miR-16a-5p abolished shcircTLK1's protective effect in neurons (Chen W. et al., 2022). Likewise, Cao et al. discovered that the expression of circ_0070441 increased in MPP+-treated SH-SY5Y cells (Cao et al., 2022). Furthermore, circ_0070441 suppresses miR-626 while increasing IRS2 expression levels through a ceRNA axis. Notably, circ_0070441 deficiency reduced MPP+-induced neuronal damage by regulating cell death and inflammation (Cao et al., 2022).

Unlike circSNCA, the circRNAs circzip-2 and circDLGAP4 have been identified to be changed in PD and to have a protective effect. On the one hand, Kumar et al. (2018) discovered circzip-2, a circRNA generated from the zip-2 gene, whose human homolog codes for CCAAT-enhancer-binding protein (C/EBP) which acted as bZIP transcription factor implicated in PD through regulating α-synuclein levels (Kumar et al., 2018; Valente et al., 2020). Cirzip-2 was predicted to sponge miR-60 using bioinformatic analysis. As a result, a reduction in circzip-2 may increase miR-60 activity, leading to downregulation of protective genes such as M60.4, ZK470.2, igeg-2, and idhg-1. On the other hand, Feng et al. (2020) reported in PD models (MPTP-induced animals and MPP+-induced cells) a reduction in the circDLGAP4 expression, which displayed neuroprotective effects *in vitro*. The authors expected that miR-134-5p might be a target for circDLGAP4 in both human and animal models, finding that this miRNA was elevated in both. Lastly, the same research established that the circDLGAP4/miR-134-5p axis influences CREB signaling and CREB downstream target gene transcription, including BDNF, Bcl-2, and PGC-1a, which are all neuroprotective proteins implicated in a variety of neurodegenerative diseases, including AD and PD (D'Orsi et al., 2017; Lv et al., 2018; Bawari et al., 2019).

### 4.8. Other ceRNAs axes

Under normal circumstances, the ceRNA axes within the cell are in equilibrium. Increasing or reducing the expression of each axis component might shift the direction in favor of abnormal circumstances (Sabaie et al., 2021). Remarkably, the majority of the remaining lncRNAs, which are the primary parts of the axes, are associated with increased expression. Xie et al. reported that SOX21-AS1 is overexpressed in MMP+-treated SH-SY5Y cells (Xie et al., 2021). In addition, SOX21-AS1 depletion weakened the cell injury induced by MMP+. Moreover, SOX21-AS1 knockdown decreased ROS generation and levels of TNF-α, IL-1β, and IL-6 but increased SOD activity. However, SOX21-AS1 up-regulation led to the opposite results. Further, SOX21-AS1 could bind with miR-7-5p, whose overexpression relieved MMP+-induced cell injury. Additionally, insulin receptor substrate 2 (IRS2) served as the target gene of miR-7-5p, and its expression was positively modulated by SOX21-AS1. Similarly, IRS2 knockdown also had alleviative effects on cell injury stimulated by MMP+ treatment (Xie et al., 2021). In this regard, Xu et al. also reported increased expression levels of ID2-AS1 in SH-SY5Y cells. Furthermore, ID2-AS1 down-regulation weakened MPP+-induced cytotoxicity in SH-SY5Y cells and alleviated the neuronal damage in PD by regulating the miR-199a-5p/IFNAR1/JAK2/STAT1 axis (Xu et al., 2021). Similarly, Zheng et al. concluded that UCA1 experienced overexpression in MPP+-induced cytotoxicity in SH-SY5Y cells, and silencing of UCA1 protects SK-N-SH cells from MPP+-evoked cytotoxicity by targeting the miR-423-5p/KCTD20 axis. The overexpression of UCA1 causes KTCD20 expression levels to increase through inhibition of miR-423-5p (Zheng et al., 2021). KCTD20 can activate the Akt signaling pathway (Nawa and Matsuoka, 2013), which plays a crucial role in the pathogenesis of PD rats and the PD neurodegeneration (Huang et al., 2018; Furlong et al., 2019). It should be noted that AL049437 is one of the lncRNAs that is overexpressed in MPP+-treated SH-SY5Y cells. Zhang et al. reported that the high levels of AL049437 reduce the expression of miR-205-5p and increase the expression of MAPK1 in SH-SY5Y cells (Zhang L. et al., 2020). MAPK1 is also involved in another ceRNA axis in PD, such as SNHG1/miR-125b-5p/MAPK1 (Xiao et al., 2021). Conversely, Zhou et al. discovered that the expression levels of NORAD as a PD protector are decreased in PD, and it was concluded that NORAD was able to up-regulate SLC5A3 with interacting miR-204-5p and protect neuroblastoma cells from MPP+-induced damage (Zhou et al., 2020a). Similarly, PART1 is a protecting factor for PD by sponging miR-106b-5p, and MCL1 is a direct target for miR-106b-5p, and consequently, PART1 reduced MPP+-induced damage to SHSY5Y cells (Shen Y. E. et al., 2021).

In a creative procedure, Feng et al. used a synthetic miRNA sponge to assess miR-330/SHIP1/NF-κB ceRNA axes. Notably, the miR-330 synthetic sponge inhibits miR-330 and suppresses LPS-induced chronic neuroinflammation in PD by down-regulating the activity of microglia with reduced inflammatory cytokines *via* the SHIP1/NF-κB signaling pathway (Feng et al., 2021). Recent research indicates that neuroinflammation, defined by abnormal activation of microglia, may play a critical role in PD (Raza et al., 2019). Remarkably, Shen et al. revealed the protective effects of another lncRNA. MIAT causes neuroprotective effects in PD by inhibiting miR-34-5p and regulating SYT1 expression. As a crucial intercessor, SYT1 governs the release of the calcium-dependent neurotransmitter and is closely linked to physiological and cognitive development (Baker et al., 2018). Local amyloid peptide buildup is thought to cause neuronal degeneration, memory impairments, synapse loss, and malfunction (Mihaescu et al., 2022). SYT1 modulates synaptic amyloid; hence it might be used to treat nervous system problems (Kuzuya et al., 2016). It should be noted that the ABCA1/miR-873/A20 ceRNA axes can be used to target neuroinflammation. Inhibition of miR-873 through increased ABCA1 may play a dual protective role in PD as it induces intracellular cholesterol homeostasis and improves neuroinflammation (Wu et al., 2017).

Conversely, Xu et al. reported the non-protective role of GAS5 in PD. GAS5 positively regulates NLRP3 by competitive sponging of miR-223-3p. In addition, GAS5 knocking down causes an increase in the expression levels of miR-233-3p, which reduces inflammatory factors (Xu et al., 2020). Similarly, Zhang et al. revealed that decreasing the expression levels of MIR17HG reduces inflammatory responses in microglia in PD models. MIR17HG experiences an increase in PD inhibits miR-153-3p, and causes an increase in SNCA, directly affecting the PD development (Zhang et al., 2022).

## 5. Limitations

The present study had several limitations. On the one hand, this study attempted to serve as a road map for future research in this field and generate interest and excitement for future ceRNA investigations in PD, and these studies will undoubtedly assist in finishing this path. On the other hand, in the section on significant results, we attempted to provide all of the details relevant to all of these regulatory axes in Table 1. All precautions were made to avoid missing a study during the screening process, and three people collaborated on this part to ensure that this study included all of the studies completed in the area of PD. However, this is possible due to an individual mistake that may have left a study out.

## 6. Conclusion

In recent years, several ceRNA axes have been found and examined in various diseases. Because ceRNA interaction networks are multifactorial, they may provide an advantage in investigations of these complex neurodegenerative diseases such as PD, both at the level of biomarkers (combined RNA biomarkers panels) and targeted therapies (regulate the multiple disease-associated RNA levels at once by just targeting one). This study collected evidence that the ceRNA axis has a remarkable influence on PD development, each of which has the potential to be a distinguishing feature of this neurodegenerative disease. The multitude of studies in the field of ceRNA axes and the results of bioinformatic analysis of the enrichment of genes targeted in ceRNA axes, including cellular response to the metal ion, cellular response to oxidative stress, and positive regulation of macromolecule metabolic processes, indicate the importance of these axes in the development of PD. The strength of these studies appears to lie in how these axes provide the ability to narrow the border between diagnosis and treatment in PD. To conclude, the upward trend of studies around ceRNA axes will lead to the evolution of ceRNET and the evolution of our knowledge of this network as one of the superior molecular mechanisms that enable the transformation of studies in the field of treatment with a unique look at ceRNA axes.

## Data availability statement

The original contributions presented in the study are included in the article/Supplementary material, further inquiries can be directed to the corresponding author.

## Author contributions

MA, SG-F, and HS wrote the draft and revised it. MT and MR designed and supervised the study. BH, SA, GK, FF, MM, and MS-B collected the data and designed the figures and tables. All the authors read and approved the submitted version.
